# Exploring the association between triglyceride glucose index-related obesity indices and asthma–COPD overlap: NHANES 2001 to 2018

**DOI:** 10.1097/MD.0000000000044294

**Published:** 2025-09-05

**Authors:** Haoran Qu, Qihang Xie, Yiyun Yang, Yue Shao, Changying Li

**Affiliations:** a Department of Cardiothoracic Surgery, The First Affiliated Hospital of Chongqing Medical University, Chongqing, China; b Department of Anesthesiology, Children’s Hospital of Chongqing Medical University, Chongqing, China.

**Keywords:** asthma–COPD overlap, insulin resistance, NHANES, TyG-related obesity index

## Abstract

The association between asthma and chronic obstructive pulmonary disease overlap (ACO) and insulin resistance (IR) has not been adequately investigated. Triglyceride glucose (TyG) index-related obesity indices offer a novel measure for assessing IR. We aimed to explore the associations between these indices and ACO in US population. Data used in this study were obtained from the National Health and Nutrition Examination Survey. We performed logistic regression analysis, restricted cubic spline modeling, subgroup analysis, sensitivity analysis, and additional analyses to examine the association between TyG-related obesity indices and ACO. The study involved 11,453 participants. TyG-waist to height ratio, TyG-body mass index, TyG-weight adjusted waist index, and TyG-waist circumference were all associated with ACO in multivariate logistic regression, with adjusted odds ratios (ORs) (95% confidence interval [CI]) of 1.23 (1.11–1.37), 1.32 (1.12–1.57), 1.20 (1.08–1.34), 1.14 (1.06–1.22), respectively. The highest quartile of all indices had the strongest link with ACO, as evidenced for TyG-waist to height ratio (OR [95% CI] = 1.80 [1.29–2.52]), TyG-body mass index (OR [95% CI] = 1.59 [1.19–2.14]), TyG-weight adjusted waist index (OR [95% CI] = 1.82 [1.23–2.69]), and TyG-waist circumference (OR [95% CI] = 1.75 [1.28–2.39]) in the fully adjusted model. Most subgroup, sensitivity, and supplementary analyses revealed similar results. TyG-related obesity indices were significantly associated with ACO. This finding indicates a strong correlation between high IR and susceptibility to ACO in the US population.

## 1. Introduction

The Global Initiative for Asthma and Global Initiative for Obstructive Lung Disease (GOLD) define asthma-chronic obstructive pulmonary disease (COPD) overlap (ACO) as a distinct clinical entity. This medical condition is characterized by ongoing airflow restriction and clinical characteristics that combine the elements of both asthma and COPD.^[1]^ The current evidence indicates that ACO is commonly encountered in clinical practice. Although the GOLD report recommended discontinuing the use of the term “ACO” in 2021, ACO has received much attention as a medical condition or characteristic.^[2]^ The incidence of ACO varies widely in different reports, and its prevalence ranges between 0.9% and 11.1% in the general population.^[1,3,4]^ A previous meta-analysis found the pooled incidence of ACO among COPD patients to be 29.6% and 6.2% in patients with asthma.^[5]^ The number of people with ACO is estimated to increase with an increase in the number of people with asthma and COPD.^[6]^ Notably, patients with ACO experience more severe symptoms, more comorbidities, poorer quality of life, higher healthcare utilization, and increased mortality than those with asthma or COPD alone.^[1,7–10]^ Therefore, early detection and intervention in ACO are crucial for controlling disease progression.

Insulin resistance (IR) has been identified to be correlated with increased morbidity, prevalence, and severity of COPD and asthma.^[11–15]^ The gold standard for evaluating IR is the use of a hyperinsulinemic–euglycemic clamp.^[16]^ However, its application in large-scale studies is limited due to its invasiveness and utility. Therefore, researchers have proposed new tools for evaluating IR, such as the homeostasis model assessment of insulin resistance (HOMA-IR), which facilitates clinical use.^[17]^ In recent years, reliable and easy tools have been devised, including the triglyceride glucose (TyG) index, which eliminates the need to measure plasma insulin levels.^[18,19]^ Researchers have suggested new indices based on the TyG termed the TyG-waist to height ratio (TyG-WHtR), TyG-body mass index (TyG-BMI), TyG-waist circumference (TyG-WC), and TyG-weight adjusted waist index (TyG-WWI), which can assess the severity of IR more accurately by combining obesity-related anthropological parameters.^[20,21]^ Further, these composite indices reportedly have strong correlations with metabolic syndrome,^[22]^ diabetes mellitus,^[23]^ and they are more accurate predictors of disease risk compared to TyG alone.^[24,25]^ However, whether IR is linked to the incidence or progression of ACO, as in asthma or COPD, has not been adequately studied.

In this cross-sectional study based on a US population, we aimed to offer some novel insights into the relationship between IR and ACO by exploring the association between TyG-related obesity indices and ACO. Given the cross-sectional nature of the National Health and Nutrition Examination Survey (NHANES) data, this study explores the associations between TyG indices and ACO but cannot infer causality.

## 2. Methods

### 2.1. Study design and participants

**Figure 1.**
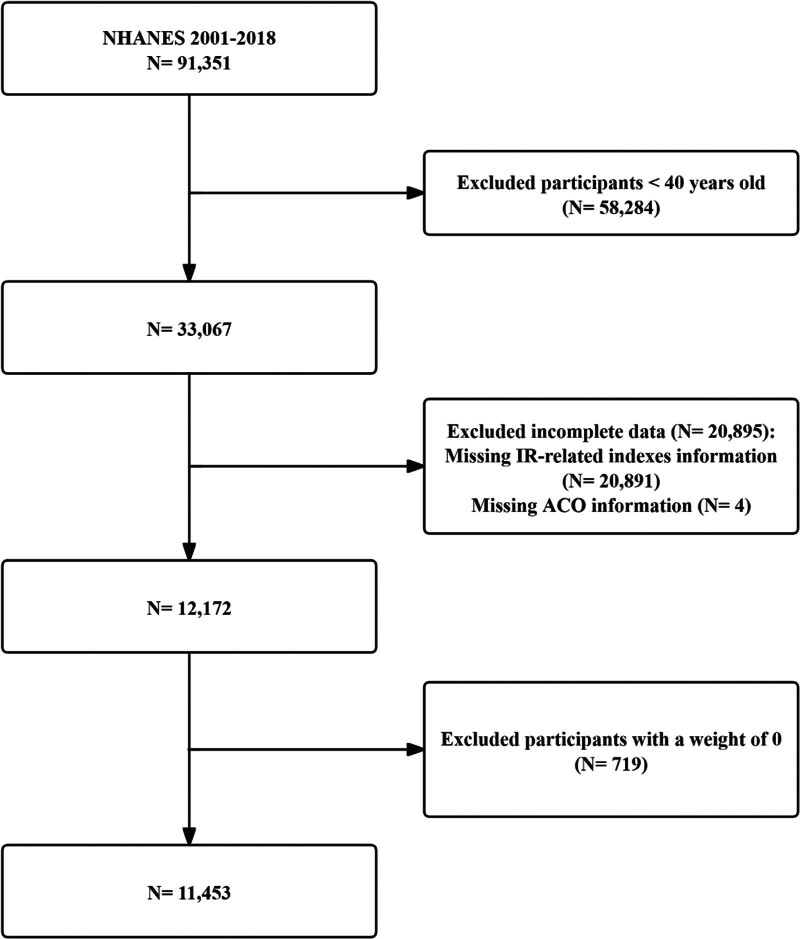
Flow chart of data collection and processing.

The data in this cross-sectional study were gathered from the NHANES, a dataset that utilizes a multi-stage, stratified probability sampling strategy to collect health, nutrition, and lifestyle data on the US population to comprehensively promote the nation’s health.^[26]^ The study dataset included participants from 9 pooled NHANES cycles for 2001 to 2018 and the following exclusion criteria were followed: age < 40 years (A widely recognized criterion for the identification of both ACO and COPD was age ≥ 40 years)^[27,28]^; missing data for calculating IR-related indices (fasting blood glucose [FBG], triglycerides [TG], high density lipoprotein cholesterol, fasting serum insulin [FINS], BMI, and WC); missing data on diagnosis of ACO; and participants with a weight of 0. Ultimately, 11,453 participants were enrolled in the analysis, of whom 655 were diagnosed with ACO. The data screening process is illustrated in Figure 1.

### 2.2. Definition and calculation of IR-related indices

TyG-related obesity indices (TyG-WHtR, TyG-BMI, TyG-WWI, and TyG-WC) served as the exposure variables, and data used for calculations were obtained from FBG, TG, and certain body parameters. Specially trained healthcare professionals conducted the anthropometric measurements, and baseline blood samples were collected to measure FBG and TG levels. The formula for the calculation was as follows: TyG = Ln [TG (mg/dL) × FBG (mg/dL)/2].^[29]^ The obesity-related TyG indices were further calculated as follows: TyG-WHtR = TyG × WHtR (WHtR = WC/height); TyG-BMI = TyG × BMI; TyG-WC = TyG × WC; and TyG-WWI = TyG × WWI (WWI = WC/$\sqrt{Weight}$).^[24,30,31]^ Traditional IR-related index in supplemental analysis: HOMA-IR = (FINS (mU/L) × FBG (mg/dL)/18)/22.5.^[32]^

### 2.3. Definitions of asthma, COPD, and ACO

Participants were considered to have asthma when they reported at least one of the following conditions^[33,34]^: self-reported physician diagnosis, recent asthma attack (past year), or wheezing symptoms (past year). Those who met at least one of the following conditions were diagnosed with COPD^[35]^: previous diagnosis of COPD, chronic bronchitis, or emphysema by a physician or healthcare professional; forced expiratory volume in 1 second/forced vital capacity < 0.70 after inhaling a bronchodilator. Participants with at least 1 COPD and asthma condition were considered to have ACO. The definitions used in this study are widely accepted and are used in other NHANES publications.^[36,37]^

### 2.4. Covariates

Confounders that potentially influenced outcomes were identified based on previous studies. The following covariates were included: sex, age (years), educational level, race, poverty to income ratio (PIR), marital status, BMI, smoker, drinking status, hypertension, diabetes, hyperlipidemia, and cardiovascular disease (CVD). Multiple imputations using chained equations were conducted to impute the missing covariate data. Details of the covariates are presented in Appendix 1, Supplemental Digital Content, https://links.lww.com/MD/P849.

### 2.5. Statistical analysis

This study followed the NHANES analytical guidelines, accounting for the complex multi-stage probability survey design and sampling weights. The specific details are available on the NHANES website. For the full analysis, we created the Fasting Subsample 2 Year Mec Weight (WTSAF2YR × 1/9), as it constituted the smallest subsample in our study. Continuous variables are described as mean ± standard deviation, and categorical variables are presented as percentages (n%). For the baseline characteristic analysis, survey-weighted linear regression was used to compare continuous variables, whereas the survey-weighted chi-square test was used to compare categorical variables. Initially, we considered all TyG-related obesity indices to be continuous variables and categorized the participants into 4 quartiles for further examination. Relationships between TyG-related obesity indices and ACO were investigated using survey-weighted multiple logistic regression analyses within a multivariate model. Quartile 1 (Q1) was used as a reference, and odds ratios (ORs) and 95% confidence intervals (CIs) were calculated for Q2, Q3, and Q4. Three adjustment models were formulated in this study. Model 1 was unadjusted; Model 2 was adjusted for age (continuous variable), sex, and race; and Model 3 was adjusted for the variables in Model 2 plus education level, marital status, PIR, smoker, drinking status, hypertension, diabetes, hyperlipidemia, and CVD. Dose–response associations between TyG-related indices and ACO were examined using restricted cubic spline regression (knot = 3) following adjustment for all covariates in Model 3. We further performed a subgroup analysis based on the fully adjusted model to explore the potential moderating effects of sex, age, race, smoker, drinking status, BMI, diabetes, hypertension, hyperlipidemia, and CVD. We further assessed the differences between the subgroups using weighted multivariate logistic regression and evaluated the interactions between the subgroups and TyG-related obesity indices using likelihood ratio tests. Sensitivity analyses utilizing unweighted data were undertaken to assess the robustness of associations independent of NHANES sampling weights. Additionally, we conducted sensitivity analyses adjusting for the use of corticosteroids and metformin to evaluate potential confounding effects due to medication usage. These medications were incorporated as covariates in supplementary multivariable logistic regression models.

Given the high values of TyG-BMI, TyG-WC, and TyG-WWI, we scaled down TyG-BMI and TyG-WC by 100 times, and TyG-WWI by 10 times before regression analysis. These adjustments preserve the original data trends. We performed a supplementary analysis to calculate the HOMA-IR and identify the OR values under different models. Because all TyG-related obesity indices and HOMA-IR generally have right-skewed distributions, these indices were transformed into natural logarithms (Ln-transformed) in the supplementary analysis.

All analyses were completed using *R* Version 4.2.2, and the Free Statistics Analysis Platform (Version 2.0, Beijing, China). All *P* values were 2-sided, and statistical significance was defined as *P* < .05.

## 3. Results

### 3.1. Baseline characteristics of the participants

Demographic characteristics are displayed in Table 1. Of 11,453 participants included in the study, 5.72% (n = 655) were identified as having ACO. The proportion of male participants was 47.59%, and that of female participants was 52.41%. The average age of participants was 57.34 ± 11.64 (mean ± SD) years, and 39.77% were older than 60 years. Compared with participants without ACO, those with ACO were older, more likely to be female, unmarried, smokers, and heavy drinkers, had higher BMI, lower income, lower education level, and were more susceptible to hypertension, diabetes, and CVD. Moreover, participants with ACO had significantly higher TyG-related obesity indices (*P* < .05).

**Table 1:** Weighted baseline characteristics by ACO.

Variables	Overall	Non-ACO	ACO	*P* value
Participants (n)	11,453	10,798	655	
Gender (%)				<.001
Male	47.59	48.14	38.79	
Female	52.41	51.86	61.21	
Age (yr), mean ± SD	57.34 ± 11.64	57.22 ± 11.65	59.31 ± 11.32	<.001
Age (%)				.006
<60	60.23	60.64	53.66	
≥60	39.77	39.36	46.34	
Race (%)				<.001
Mexican American	6.19	6.47	1.69	
Other Hispanic	4.39	4.45	3.46	
Non-Hispanic White	72.51	72.11	79.03	
Non-Hispanic Black	10.15	10.21	9.22	
Other Races	6.76	6.77	6.60	
PIR (%)				<.001
Low income	18.20	17.52	29.08	
Medium income	35.54	35.30	39.43	
High income	46.26	47.18	31.49	
Marital status (%)				<.001
Unmarried	35.65	35.07	45.03	
Married	64.35	64.93	54.97	
Education level (%)				.001
Less than high school	17.15	16.77	23.18	
High school or equivalent	24.32	24.24	25.55	
College or above	58.53	58.99	51.27	
BMI (%)				.007
<25	26.37	26.61	22.65	
25–29.9	35.17	35.41	31.18	
≥30	38.46	37.98	46.17	
Smoker (%)				<.001
Never	50.74	52.28	26.09	
Former	30.82	30.63	33.91	
Current	18.44	17.09	39.99	
Drinking status (%)				<.001
Never	11.28	11.49	7.95	
Former	40.98	41.42	34.00	
Mild	15.21	15.24	14.76	
Moderate	14.64	14.48	17.12	
Heavy	17.90	17.38	26.17	
Hypertension (%)				<.001
No	56.96	57.90	41.80	
Yes	43.04	42.10	58.20	
Diabetes (%)				.001
No	86.79	87.12	81.56	
Yes	13.21	12.88	18.44	
Hyperlipidemia (%)				.480
No	30.10	30.20	28.49	
Yes	69.90	69.80	71.51	
CVD (%)				<.001
No	86.93	88.03	69.35	
Yes	13.07	11.97	30.65	
TyG-WHtR, mean ± SD	5.27 ± 1.01	5.25 ± 1.00	5.57 ± 1.12	<.001
TyG-WWI, mean ± SD	97.44 ± 11.22	97.20 ± 11.14	101.31 ± 11.83	<.001
TyG-BMI, mean ± SD	256.28 ± 64.67	255.45 ± 63.72	269.64 ± 77.26	<.001
TyG-WC, mean ± SD	886.14 ± 170.94	883.44 ± 169.24	929.41 ± 191.15	<.001

ACO = asthma–COPD overlap, BMI = body mass index, CVD = cardiovascular disease, PIR = income-to-poverty ratio, SD = standard deviation, TyG-BMI = triglyceride glucose-body mass index, TyG-WC = triglyceride glucose-waist circumference, TyG-WHtR = triglyceride glucose-waist to height ratio, TyG-WWI = triglyceride glucose-weight-adjusted waist index.

### 3.2. Association between TyG-related obesity indicators and ACO

The relationships between TyG-related obesity indices and ACO are shown in Table 2. All indices (continuous) were positively and significantly (*P* < .05) associated with ACO, maintaining significance in both the partially and fully adjusted models. Furthermore, TyG-related obesity indices were analyzed as categorical data by dividing the cohort into quartiles (Q1–Q4). Compared to Q1, Q4 of all indices showed a positive, significant correlation with ACO, and tests for trends were significant in each model (*P* for trend < .05). This association remained significant in all 3 different adjusted models: Model 1 (TyG-WHtR: OR = 2.25; 95% CI = 1.66–3.04, TyG-BMI: OR = 1.63; 95% CI = 1.25–2.11, TyG-WWI: OR = 2.66; 95% CI = 1.93–3.66, TyG-WC: OR = 1.96; 95% CI = 1.48–2.60), Model 2 (TyG-WHtR: OR = 2.27; 95% CI = 1.67–3.07, TyG-BMI: OR = 1.77; 95% CI = 1.35–2.32, TyG-WWI: OR = 2.67; 95% CI = 1.93–3.73, TyG-WC: OR = 2.24; 95% CI = 1.66–3.03), Model 3 (TyG-WHtR: OR = 1.80; 95% CI = 1.29–2.52, TyG-BMI: OR = 1.59; 95% CI = 1.19–2.14, TyG-WWI: OR = 1.82; 95% CI = 1.23–2.69, TyG-WC: OR = 1.75; 95% CI = 1.28–2.39).

**Table 2:** Associations between TyG-related obesity indices and ACO.

Characteristic	Model 1 OR (95% CI)	*P* value	Model 2 OR (95% CI)	*P* value	Model 3 OR (95% CI)	*P* value
TyG-WHtR continuous	1.34 (1.22–1.47)	<.001	1.34 (1.22–1.47)	<.001	1.23 (1.11–1.37)	<.001
TyG-WHtR quantiles						
Q1	Reference		Reference		Reference	
Q2	1.19 (0.84–1.68)	.325	1.23 (0.87–1.74)	.250	1.18 (0.84–1.67)	.338
Q3	1.31 (0.91–1.88)	.146	1.34 (0.93–1.95)	.118	1.25 (0.87–1.79)	.234
Q4	2.25 (1.66–3.04)	<.001	2.27 (1.67–3.07)	<.001	1.80 (1.29–2.52)	<.001
*P* for trend	<.001		<.001		<.001	
TyG-BMI continuous	1.37 (1.18–1.59)	<.001	1.41 (1.21–1.64)	<.001	1.32 (1.12–1.57)	.001
TyG-BMI quantiles						
Q1	Reference		Reference		Reference	
Q2	0.87 (0.62–1.23)	.428	0.93 (0.65–1.33)	.709	0.98 (0.69–1.40)	.925
Q3	0.89 (0.64–1.23)	.466	0.96 (0.69–1.35)	.830	0.93 (0.67–1.30)	.679
Q4	1.63 (1.25–2.11)	<.001	1.77 (1.35–2.32)	<.001	1.59 (1.19–2.14)	.002
*P* for trend	<.001		<.001		.004	
TyG-WWI continuous	1.36 (1.26–1.47)	<.001	1.36 (1.26–1.47)	<.001	1.20 (1.08–1.34)	<.001
TyG-WWI quantiles						
Q1	Reference		Reference		Reference	
Q2	1.46 (1.01–2.11)	.042	1.48 (1.02–2.15)	.037	1.30 (0.89–1.90)	.176
Q3	1.48 (1.02–2.16)	.039	1.52 (1.03–2.23)	.036	1.27 (0.85–1.89)	.235
Q4	2.66 (1.93–3.66)	<.001	2.67 (1.91–3.73)	<.001	1.82 (1.23–2.69)	.003
*P* for trend	<.001		<.001		.002	
TyG-WC continuous	1.16 (1.09–1.23)	<.001	1.19 (1.12–1.26)	<.001	1.14 (1.06–1.22)	<.001
TyG-WC quantiles						
Q1	Reference		Reference		Reference	
Q2	1.20 (0.87–1.65)	.276	1.30 (0.92–1.82)	.131	1.24 (0.89–1.73)	.201
Q3	1.41 (1.01–1.97)	.041	1.61 (1.12–2.29)	.010	1.50 (1.06–2.11)	.021
Q4	1.96 (1.48–2.60)	<.001	2.24 (1.66–3.03)	<.001	1.75 (1.28–2.39)	<.001
*P* for trend	<.001		<.001		<.001	

Model 1 adjust for: None.

Model 2 adjust for: age, gender, race.

Model 3 adjust for: age, gender, race, education level, marital status, PIR, smoker, drinking status, hypertension, diabetes, hyperlipidemia, and CVD.

95% CI = 95% confidence intervals, OR = odds ratios, TyG-BMI = triglyceride glucose-body mass index, TyG-WC = triglyceride glucose-waist circumference, TyG-WHtR = triglyceride glucose-waist to height ratio, TyG-WWI = triglyceride glucose-weight-adjusted waist index.

**Figure 2.**
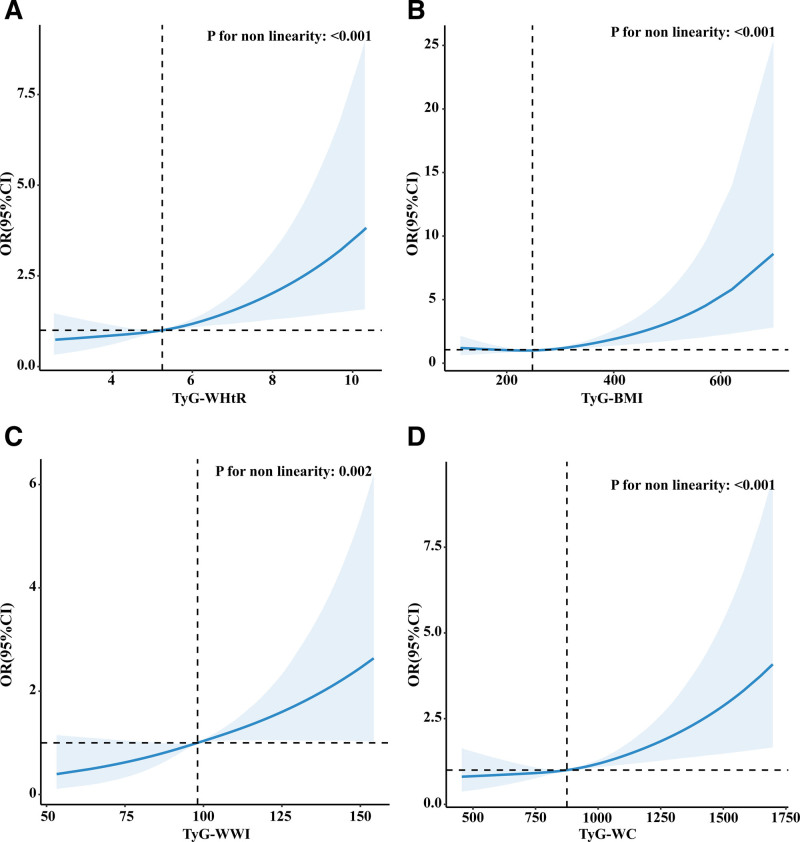
Examination of the dose–response relationship between TyG-related obesity indices and ACO, analyzed using restricted cubic spline (RCS) with adjustments for covariates in model 3. (A) TyG-WHtR; (B) TyG-BMI; (C) TyG-WWI; (D) TyG-WC. ACO = asthma and chronic obstructive pulmonary disease overlap, BMI = body mass index, TyG = triglyceride glucose, WC = waist circumference, WHtR = waist to height ratio, WWI = weight adjusted waist index.

Subsequently, we conducted a restricted cubic spline analysis, which was adjusted for all covariates in Model 3. Figure 2 illustrates a positive non-linear relationship between TyG-WHtR, TyG-WWI, TyG-WC, and ACO (non-linear *P* < .05). A non-linear relationship between TyG-BMI and ACO was also observed (non-linear *P* < .001).

### 3.3. Subgroup analysis

**Figure 3.**
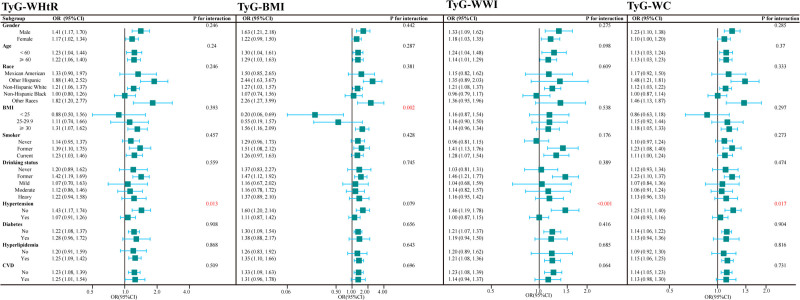
Subgroup analysis of the association between TyG-WHtR, TyG-BMI, TyG-WWI, TyG-WC and ACO. Except for the stratification component, each stratification factor was adjusted for Model 3. ACO = asthma and chronic obstructive pulmonary disease overlap, BMI = body mass index, TyG = triglyceride glucose, WC = waist circumference, WHtR = waist to height ratio, WWI = weight adjusted waist index.

Subgroup analyses were performed to explore the association between TyG-related obesity indices and ACO (Fig. 3). All the covariates in Model 3 were considered, except for the variable used to create the subgroup. The relationship between TyG-related obesity indices and ACO was not affected by most stratification variables (interaction, *P* > .05). Significant interaction effects were observed among TyG-WHtR, TyG-WC, TyG-WWI, and ACO in the hypertension subgroup (interaction, *P* < .05). Additionally, BMI was found to modulate the link between TyG-BMI and ACO, with a more pronounced association observed in the subgroup with a higher BMI (interaction, *P* = .002).

### 3.4. Sensitivity analysis

As shown in Table S1, Supplemental Digital Content, https://links.lww.com/MD/P849, we conducted sensitivity analyses using unweighted multivariable logistic regression and found that TyG-related indices were positively associated with ACO in all models (*P* < .05), whether analyzed as continuous variables or quartiles. These findings are consistent with those of the survey-weighted analysis presented in Table 2. The associations between TyG-related indices and ACO remained robust after adjusting for corticosteroid and metformin use. Detailed results are presented in Table S2, Supplemental Digital Content, https://links.lww.com/MD/P849.

### 3.5. Supplementary analysis

To further examine the representativeness of TyG-related obesity indices, we performed an additional analysis to compare these indices with the traditional IR-related index (HOMA-IR). The results showed that all TyG-related obesity indices demonstrated more significant associations with ACO compared to HOMA-IR (Table S3, Supplemental Digital Content, https://links.lww.com/MD/P849).

## 4. Discussion

In this nationwide cross-sectional study, we explored the correlation between TyG-related obesity indices and ACO using the NHANES database for 2001 to 2018. Our results provide new evidence for the correlation between IR and ACO. The predominant findings suggest that TyG-WHtR, TyG-BMI, TyG-WWI, and TyG-WC are positively associated with the incidence of ACO in the US population. These correlations remained significant after adjusting for all covariates (sex, age, PIR, educational level, smoker, drinking status, marital status, hypertension, hyperlipidemia, diabetes, CVD). Further analysis showed that this correlation was robust across nearly all subsets, and associations between TyG-related obesity indices and ACO were stronger than the traditional IR index (HOMA-IR) according to the supplemental analysis. These indices can be easily assessed in clinical practice, which is important for the early detection and intervention of ACO to reduce the disease burden. Future longitudinal cohort studies should validate whether baseline TyG-related obesity indices predict incident ACO and refine clinically relevant thresholds.

The relationship between IR and chronic respiratory diseases has been extensively investigated. A recent study from the Korea National Health and Nutrition Examination Survey^[38]^ indicated a positive correlation among insulin levels, diabetes, and prevalent asthma. Epidemiological cohort studies have demonstrated an association between IR and onset of asthma and asthma-like symptoms.^[11]^ Several studies have shown that IR contributes to COPD progression.^[39]^ IR-related indices have also been used to assess the relationship between respiratory diseases and IR. Wu et al assessed the TyG index in relation to pulmonary health and provided recent evidence for an association between the TyG index and respiratory symptoms, restrictive spirometry patterns, and chronic bronchitis.^[40]^ To the best of our knowledge, the relationship between IR and ACO has not been extensively studied. Our results provide new evidence for an association between IR and ACO.

HOMA-IR is a classical IR index that has been used in many studies and is based on FBG and FINS levels.^[17]^ However, considering the high cost of insulin detection and the limitations of testing patients undergoing insulin therapy, a more efficient, convenient, and representative index is required. In recent years, the TyG index has become a reliable surrogate marker of IR because of its specificity, simplicity, and sensitivity.^[41]^ Previous research has demonstrated its superiority over HOMA-IR in identifying IR.^[24]^ Based on the TyG, novel TyG-related obesity indices have been derived by combining anthropometric and metabolic indices, namely TyG-WHtR, TyG-BMI, TyG-WWI, and TyG-WC. TyG-related obesity indices have been extensively studied for their applicability to various clinical diseases.^[22,24,25]^ Combining obesity metrics with TyG could enhance the prediction of metabolic syndrome risk compared with using these indices alone.^[42]^ Additionally, TyG-related indices demonstrated greater robustness in predicting IR than TyG alone in the general population.^[24]^ Therefore, the integration of obesity indices with TyG may more precisely represent the relationship between IR and ACO, providing a rationale for the selection of these indices in the present study.

At present, the pathophysiology of the relationship between TyG-related obesity indices and ACO remains unknown, but several possible factors may account for this relationship. A joint statement published by the Global Initiative for Asthma and GOLD in 2014 defined ACO as a medical condition characterized by persistent airflow limitation and characteristics of asthma and COPD.^[1]^ Emerging longitudinal evidence supports a detrimental role of IR in lung health. A South Korean cohort^[38]^ and analyses from the Severe Asthma Research Program-3 cohort (5-year follow-up)^[43]^ both demonstrate that IR accelerates lung function decline. In addition, a high insulin concentration could inhibit muscarinic M2 receptors on pulmonary parasympathetic nerves in both humans and mice to increase airway hyperresponsiveness^[12]^ and can also directly contribute to endothelial cell damage and the proliferation of airway smooth muscle cells, potentially resulting in airway narrowing.^[44]^ According to the results of Park, YH et al, expression of TGF-β1 is upregulated in the bronchial epithelium due to IR, which may be essential for airway hyperresponsiveness and development of lung fibrosis. Chronic airway inflammation is one of the main factors associated with ACO.^[45]^ One study reported that IR could induce airway inflammation by negatively regulating Sfrp5 (an anti-inflammatory adipocytokine) expression and activating the Wnt5a/JNK1 pathway.^[46]^ Additionally, IR could elevate bacterial colonization risk of the airways and enhance the production of pro-inflammatory mediators (interleukin-6 and tumor necrosis factor-alpha) from adipose tissue, exacerbating Th2 inflammation.^[13]^ Numerous studies in humans and animal models have confirmed that IR is associated with reduced mitochondrial mass or oxidative function in insulin-sensitive tissues,^[47]^ mitochondrial dysfunction is a common link between obesity, metabolic syndrome, and asthma.^[48]^ The combined effect of these mechanisms may explain the association between IR and a high prevalence of ACO. Further studies are required to investigate the potential biological mechanisms underlying the association and search for new therapeutic targets.

Notably, our findings indicate that the observed association was more pronounced in the non-hypertensive population. This finding suggests that healthcare providers should focus on the degree of IR in the non-hypertensive population; however, this result needs to be interpreted cautiously, as it may be limited by the small sample size. Future large-scale multicenter prospective studies are required to confirm our findings.

The present study had several strengths. First, the dataset was retrieved from the NHANES and Nutrition Examination Survey. Second, all analyses were adjusted for confounding factors that may be related to both the exposure and outcome variables, which enhanced the robustness of the results. Finally, our study comprehensively assessed the correlation between TyG-related obesity indicators and ACO, thus filling a clear research gap.

This study has some limitations. First, the cross-sectional design could not explain the causal relationship between the TyG-related obesity indices and ACO. Second, the diagnoses of asthma, COPD, and ACO were mainly based on a questionnaire survey owing to the absence of laboratory data related to respiratory function, coupled with the absence of precise diagnostic information; the inevitable selection bias may also reduce the robustness of the results. Finally, despite adjusting for multiple covariates, the potential for residual confounding persisted. Further large-scale, well-designed prospective studies are warranted to validate these results.

## 5. Conclusion

The main observation of this study was that the TyG-WHtR, TyG-BMI, TyG-WWI, and TyG-WC were strongly associated with the prevalence of ACO in the US population. This illustrates that TyG-related obesity indices have great potential for identifying ACO risk and that IR may play a significant role in the pathogenesis of ACO. These findings indicate that TyG-related obesity indices, as low-cost metabolic biomarkers, may aid in identifying individuals at risk of ACO for prioritized pulmonary evaluation. Future studies should validate their predictive utility in longitudinal cohorts.

## Acknowledgments

We thank all *NHANES* participants and staff for their contributions to the project.

## Author contributions

**Conceptualization:** Haoran Qu, Yue Shao, Changying Li.

**Data curation:** Haoran Qu, Qihang Xie.

**Formal analysis:** Haoran Qu, Qihang Xie, Yiyun Yang.

**Methodology:** Yue Shao, Changying Li.

**Project administration:** Yue Shao, Changying Li.

**Validation:** Haoran Qu, Qihang Xie, Yue Shao, Changying Li.

**Visualization:** Haoran Qu, Yiyun Yang.

**Writing – original draft:** Haoran Qu, Changying Li.

**Writing – review & editing:** Qihang Xie, Yue Shao.

## Supplementary Material


