# Supplementary material for: Exploring the association between triglyceride glucose index-related obesity indices and asthma–COPD overlap: NHANES 2001 to 2018
Source: Medicine (Baltimore). 2025 Sep 5;104(36):e44294. doi: 10.1097/MD.0000000000044294 (PMC12419257; doi:10.1097/MD.0000000000044294)
Supplement: Supplementary file 1 [file medi-104-e44294-s001.docx]

**Supplementary Material**

**Appendix 1.** Definition of covariates

**Education level:** divided into three categories: Less than high school, High school or equivalent, and College or above;

**Race:** included five categories: Mexican America, Other Hispanic, Non-Hispanic White, Non-Hispanic Black, and Other Races;

**PIR:** categorized as ≤1.3 for low income, 1.3-3.5 for medium income, and >3.5 for high income^1^;

**Smoker:** Smoker was grouped into “never” (never smoked or less than a hundred cigarettes in life), “current” (more than a hundred cigarettes in life and is also ongoing currently), or “former” (more than a hundred cigarettes in life but currently not smoking);

**Drinking status:** First, people who had <12 drinks in their lifetime were defined as “never” alcohol drinkers. “Former” drinkers were those who did not drink alcohol last year but had drunk ≥12 drinks in their lifetime previously. Among those who drank alcohol currently, men who had ≥4 drinks per day or women who had ≥3 drinks per day were regarded as “heavy” drinkers, while men who had 3 drinks per day or women who had 2 drinks per day were “moderate” drinkers. The remaining population was defined as “mild” drinkers. “Heavy” drinkers also contained those who had been binge drinking ≥5 days per month, and those who were binge drinking ≥2 days per month were defined as “moderate” drinkers^2^;

**Hypertension:** Hypertension was defined as a systolic blood pressure ≥ 140 mmHg or diastolic blood pressure ≥ 90 mmHg, or a self-reported history of hypertension or oral antihypertensive medications^3^;

**Diabetes:** Diabetes was defined by self-reported diagnosis, use of insulin or oral hypoglycemic medication, FBG ≥126 mg/dl, OGTT ≥200 mg/dl, or HbA1c ≥6.5%.

**Hyperlipidemia:** Hyperlipidemia was defined as having total cholesterol ≥200 mg/dl, TG ≥150 mg/dl, HDL-C < 40 mg/dl in males and < 50 mg/dl in females, or low-density lipoprotein cholesterol ≥130 mg/dl^4^;

**Cardiovascular disease (CVD):** Cardiovascular disease (CVD) cases were identified through self-reported physician diagnoses, including congestive heart failure, coronary heart disease, angina, heart attack, or stroke.

**Table S1** unweighted multivariable logistic regression analysis of TyG-related obesity indices with ACO

| **Characteristic** | | **Model 1**  **OR (95%CI)** | **Model 2**  **OR (95%CI)** | **Model 3**  **OR (95%CI)** |
| --- | --- | --- | --- | --- |
| TyG-WHtR continuous | | 1.40 (1.30, 1.51) | 1.40 (1.30, 1.51) | 1.27 (1.16, 1.39) |
| TyG-WHtR quantiles | |  |  |  |
| Q1 |  | Reference | Reference | Reference |
| Q2 |  | 1.13 (0.87, 1.46) | 1.16 (0.89, 1.51) | 1.18 (0.84, 1.67) |
| Q3 |  | 1.45 (1.13, 1.85) | 1.40 (1.07, 1.81) | 1.25 (0.87, 1.79) |
| Q4 |  | 2.32 (1.84, 2.91) | 1.87 (1.44, 2.43) | 1.80 (1.29, 2.52) |
| P for trend | | <0.001 | <0.001 | <0.001 |
| TyG-BMI continuous | | 1.49 (1.33, 1.66) | 1.52 (1.36, 1.71) | 1.37 (1.21, 1.56) |
| TyG-BMI quantiles | |  |  |  |
| Q1 |  | Reference | Reference | Reference |
| Q2 |  | 0.82 (0.64, 1.05) | 0.88 (0.69, 1.13) | 0.90 (0.69, 1.16) |
| Q3 |  | 0.99 (0.78, 1.26) | 1.07 (0.84, 1.36) | 1.03 (0.80, 1.33) |
| Q4 |  | 1.80 (1.45, 2.22) | 1.95 (1.57, 2.43) | 1.67 (1.31, 2.13) |
| P for trend | | <0.001 | <0.001 | <0.001 |
| TyG-WWI continuous | | 1.34 (1.26, 1.44) | 1.37 (1.28, 1.47) | 1.21 (1.11, 1.32) |
| TyG-WWI quantiles | |  |  |  |
| Q1 |  | Reference | Reference | Reference |
| Q2 |  | 1.65 (1.28, 2.13) | 1.73 (1.34, 2.25) | 1.54 (1.18, 2.01) |
| Q3 |  | 1.48 (1.14, 1.92) | 1.59 (1.22, 2.08) | 1.31 (0.99, 1.74) |
| Q4 |  | 2.69 (2.12, 3.41) | 2.91 (2.26, 3.73) | 2.01 (1.51, 2.68) |
| P for trend | | <0.001 | <0.001 | <0.001 |
| TyG-WC continuous | | 1.21 (1.16, 1.27) | 1.23 (1.17, 1.29) | 1.16 (1.10, 1.22) |
| TyG-WC quantiles | |  |  |  |
| Q1 |  | Reference | Reference | Reference |
| Q2 |  | 1.25 (0.97, 1.62) | 1.35 (1.04, 1.76) | 1.30 (0.99, 1.70) |
| Q3 |  | 1.63 (1.27, 2.09) | 1.79 (1.4, 2.31) | 1.64 (1.26, 2.13) |
| Q4 |  | 2.34 (1.86, 2.96) | 2.55 (2.01, 3.24) | 1.97 (1.51, 2.57) |
| P for trend | | <0.001 | <0.001 | <0.001 |

Model 1 adjusted by: None

Model 2 adjusted by: age, gender, race

Model 3 adjusted by: age, gender, race, education level, marital status, PIR, smoker, drinking status, hypertension, diabetes, hyperlipidemia, and CVD

Abbreviations: OR, odds ratios; 95% CI, 95% confidence intervals; TyG-WHtR, triglyceride glucose-waist to height ratio; TyG-BMI, triglyceride glucose-body mass index; TyG-WWI, triglyceride glucose-weight-adjusted waist index; TyG-WC, triglyceride glucose-waist circumference.

**Table S2** Associations between TyG-related obesity indices and ACO in multivariable models further adjusted for Corticosteroids and Metformin Use (N=11 448).

| **Characteristic** | **Model 1**  **OR (95%CI)** | **P value** | **Model 2**  **OR (95%CI)** | **P value** | **Model 4**  **OR (95%CI)** | **P value** |
| --- | --- | --- | --- | --- | --- | --- |
| TyG-WHtR continuous | 1.34 (1.22, 1.47) | <0.001 | 1.34 (1.22, 1.47) | <0.001 | 1.21 (1.08, 1.36) | <0.001 |
| TyG-WHtR quantiles | |  |  |  |  |  |
| Q1 | Reference |  | Reference |  | Reference |  |
| Q2 | 1.19 (0.84, 1.68) | 0.325 | 1.23 (0.87, 1.74) | 0.250 | 1.22 (0.85, 1.76) | 0.277 |
| Q3 | 1.31 (0.91, 1.88) | 0.143 | 1.35 (0.93, 1.96) | 0.115 | 1.30 (0.90, 1.88) | 0.156 |
| Q4 | 2.25 (1.66, 3.05) | <0.001 | 2.27 (1.68, 3.08) | <0.001 | 1.76 (1.25, 2.49) | 0.002 |
| P for trend | <0.001 |  | <0.001 |  | 0.001 |  |
| TyG-BMI continuous | 1.37 (1.18, 1.59) | <0.001 | 1.41 (1.22, 1.64) | <0.001 | 1.31 (1.10, 1.56) | 0.003 |
| TyG-BMI quantiles | |  |  |  |  |  |
| Q1 | Reference |  | Reference |  | Reference |  |
| Q2 | 0.87 (0.62, 1.23) | 0.428 | 0.93 (0.65, 1.33) | 0.708 | 1.02 (0.70, 1.47) | 0.935 |
| Q3 | 0.89 (0.64, 1.23) | 0.470 | 0.97 (0.69, 1.35) | 0.834 | 0.96 (0.68, 1.36) | 0.812 |
| Q4 | 1.63 (1.25, 2.12) | <0.001 | 1.78 (1.35, 2.33) | <0.001 | 1.58 (1.15, 2.16) | 0.005 |
| P for trend | <0.001 |  | <0.001 |  | 0.007 |  |
| TyG-WWI continuous | 1.36 (1.26, 1.47) | <0.001 | 1.36 (1.26, 1.47) | <0.001 | 1.18 (1.06, 1.32) | 0.003 |
| TyG-WWI quantiles | |  |  |  |  |  |
| Q1 | Reference |  | Reference |  | Reference |  |
| Q2 | 1.47 (1.01, 2.12) | 0.042 | 1.49 (1.03, 2.15) | 0.037 | 1.32 (0.90, 1.94) | 0.158 |
| Q3 | 1.48 (1.02, 2.16) | 0.039 | 1.52 (1.03, 2.24) | 0.036 | 1.27 (0.85, 1.89) | 0.246 |
| Q4 | 2.67 (1.94, 3.67) | <0.001 | 2.68 (1.92, 3.74) | <0.001 | 1.74 (1.17, 2.57) | 0.006 |
| P for trend | <0.001 |  | <0.001 |  | 0.005 |  |
| TyG-WC continuous | 1.16 (1.09, 1.23) | <0.001 | 1.19 (1.12, 1.26) | <0.001 | 1.13 (1.05, 1.21) | 0.001 |
| TyG-WC quantiles | |  |  |  |  |  |
| Q1 | Reference |  | Reference |  | Reference |  |
| Q2 | 1.20 (0.87, 1.65) | 0.276 | 1.30 (0.92, 1.82) | 0.131 | 1.29 (0.91, 1.81) | 0.146 |
| Q3 | 1.42 (1.02, 1.98) | 0.040 | 1.61 (1.13, 2.30) | 0.009 | 1.56 (1.11, 2.18) | 0.011 |
| Q4 | 1.97 (1.48, 2.60) | <0.001 | 2.24 (1.66, 3.03) | <0.001 | 1.76 (1.27, 2.43) | <0.001 |
| P for trend | <0.001 |  | <0.001 |  | <0.001 |  |

Model 1 adjusted by: None

Model 2 adjusted by: age, gender, race

Model 4 adjusted by: age, gender, race, education level, marital status, PIR, smoker, drinking status, hypertension, diabetes, hyperlipidemia, CVD, corticosteroids use, metformin use.

Abbreviations: OR, odds ratios; 95% CI, 95% confidence intervals; TyG-WHtR, triglyceride glucose-waist to height ratio; TyG-BMI, triglyceride glucose-body mass index; TyG-WWI, triglyceride glucose-weight-adjusted waist index; TyG-WC, triglyceride glucose-waist circumference.

**Table S3.** Multivariate logistic regression analysis of TyG-related obesity indices, HOMA-IR with ACO in supplementary analysis.

| **Characteristic** | **Model 1**  **OR (95%CI)** | **P value** | **Model 2**  **OR (95%CI)** | **P value** | **Model 3**  **OR (95%CI)** | **P value** |
| --- | --- | --- | --- | --- | --- | --- |
| HOMA-IR | 1.23 (1.08, 1.40) | 0.002 | 1.28 (1.12, 1.47) | <0.001 | 1.19 (1.04, 1.36) | 0.012 |
| TyG-WHtR | 4.86 (2.78, 8.50) | <0.001 | 4.85 (2.80, 8.40) | <0.001 | 3.05 (1.64, 5.68) | <0.001 |
| TyG-BMI | 2.07 (1.32, 3.27) | 0.002 | 2.31 (1.47, 3.63) | <0.001 | 1.92 (1.20, 3.08) | 0.007 |
| TyG-WWI | 23.29 (10.40, 52.15) | <0.001 | 23.28 (10.23, 53.00) | <0.001 | 6.58 (2.22, 19.53) | <0.001 |
| TyG-WC | 3.66 (2.05, 6.55) | <0.001 | 4.70 (2.59, 8.53) | <0.001 | 2.99 (1.57, 5.71) | 0.001 |

Model 1 adjusted by: None; Model 2 adjusted by: age, gender, race; Model 3 adjusted by: age, gender, race, education level, marital status, PIR, smoking status, drinking status, hypertension, diabetes, hyperlipidemia, and CVD.

Abbreviation: OR, odds ratios; 95% CI, 95% confidence intervals; TyG-WHtR, triglyceride glucose-waist to height ratio; TyG-BMI, triglyceride glucose-body mass index; TyG-WWI, triglyceride glucose-weight-adjusted waist index; TyG-WC, triglyceride glucose-waist circumference; HOMA-IR, the homeostasis model assessment of insulin resistance.

**References:**

**1.** Shen R, Lin L, Bin Z, Qiao X. The U-shape relationship between insulin resistance-related indexes and chronic kidney disease: a retrospective cohort study from National Health and Nutrition Examination Survey 2007-2016. *Diabetol Metab Syndr.* 2024;16(1):168.

**2.** Lai B, Jiang H, Gao R, Zhou X. Association between alcohol intake and bone mineral density: results from the NHANES 2005-2020 and two-sample Mendelian randomization. *Arch Osteoporos.* 2024;19(1):21.

**3.** Zeng J, Zhang T, Yang Y, et al. Association between a metabolic score for insulin resistance and hypertension: results from National Health and Nutrition Examination Survey 2007-2016 analyses. *Front Endocrinol (Lausanne).* 2024;15:1369600.

**4.** ElSayed NA, Aleppo G, Aroda VR, et al. 2. Classification and Diagnosis of Diabetes: Standards of Care in Diabetes-2023. *Diabetes Care.* 2023;46(Suppl 1):S19-S40.
